# Diagnosis of Human Leptospirosis in a Clinical Setting: Real-Time PCR High Resolution Melting Analysis for Detection of *Leptospira* at the Onset of Disease

**DOI:** 10.1038/s41598-018-27555-2

**Published:** 2018-06-15

**Authors:** Lisa M. Esteves, Sara M. Bulhões, Claudia C. Branco, Teresa Carreira, Maria L. Vieira, Maria Gomes-Solecki, Luisa Mota-Vieira

**Affiliations:** 1 Molecular Genetics and Pathology Unit, Hospital of Divino Espírito Santo of Ponta Delgada, EPER, São Miguel Island, Azores, Portugal; 2 Azores Genetics Research Group, Instituto Gulbenkian de Ciência, Oeiras, Portugal; 3 Faculty of Sciences, BioISI - Biosystems & Integrative Sciences Institute, University of Lisboa, Campo Grande, C8 bdg, 1749-016 Lisboa, Portugal; 4 Global Health and Tropical Medicine (GHTM), Instituto de Higiene e Medicina Tropical (IHMT), Universidade Nova de Lisboa (UNL), Lisboa, Portugal; 5 Department of Microbiology, Immunology and Biochemistry, The University of Tennessee Health Science Center, Memphis, Tennessee USA

## Abstract

Currently, direct detection of *Leptospira* can be done in clinical laboratories by conventional and by real-time PCR (qRT-PCR). We tested a biobank of paired samples of serum and urine from the same patient (202 patients) presenting at the hospital in an area endemic for leptospirosis using qRT-PCR followed by high resolution melting (HRM) analysis. The results were compared with those obtained by conventional nested PCR and with the serologic gold standard microscopic agglutination test (MAT). Differences were resolved by sequencing. qRT-PCR-HRM was positive for 46 of the 202 patients (22.7%, accuracy 100%) which is consistent with known prevalence of leptospirosis in the Azores. MAT results were positive for 3 of the 46 patients (6.5%). Analysis of paired samples allowed us to identify the illness point at which patients presented at the hospital: onset, dissemination or excretion. The melting curve analysis of *Leptospira* species revealed that 60.9% (28/46) of patients were infected with *L*. *interrogans* and 39.1% (18/46) were infected with *L*. *borgpetersenii*, both endemic to the Azores. We validated the use of qRT-PCR-HRM for diagnosis of leptospirosis and for identification of the *Leptospira* species at the earliest onset of infection in a clinical setting, in less than 2 hours.

## Introduction

Leptospirosis is a worldwide zoonotic and neglected infectious disease caused by pathogenic bacteria of the *Leptospira* genus from the family Leptospiraceae^1^. This disease is known for its endemicity mainly in countries with a humid tropical or subtropical climate^2^. The infection is associated with a variety of clinical manifestations, ranging from flu-like symptoms to multiple organ failure and death^3^. Symptoms of leptospirosis are frequently mistaken for other causes of acute febrile syndrome, such as dengue, hepatitis and malaria, depending on overlap of endemic geographic areas. The lack of pathognomonic signs of leptospirosis means that diagnosis is tentatively based on evaluation of fever and myalgia in patients presenting at the hospital in areas of endemicity, and it is rarely confirmed in most parts of the world due to lack of affordable diagnostic assays. As a result, laboratory support is essential.

Current techniques to detect *Leptospira* evolved from conventional to real-time PCR (qRT-PCR)^3^. An emerging technique for clinical diagnosis is high resolution melting (HRM) analysis. HRM uses affordable SYBR green chemistries and is performed with a real-time PCR instrument immediately after PCR; its underlying principle is the generation of different melting curve profiles due to sequence variations in double-stranded DNA^4^. Advantages of this method include a rapid turn-around time (less than 2 hr), a closed-tube format that significantly reduces contamination risk, high sensitivity and specificity, low cost and, unlike other methods, no sample processing or separations after PCR^5^. Furthermore, HRM is a non-damaging method that enables the subsequent analysis of the sample by other methods, such as DNA sequencing or gel electrophoresis^6^.

HRM has been used for detection of oncogene mutations^7^, human malaria diagnosis^8^, species differentiation and genotyping within microbial species^9^. For leptospirosis, several studies have described HRM for typing *Leptospira* strains at the species and subspecies levels^10–13^, mainly from *Leptospira* culture. HRM can accurately discriminate *L*. *interrogans*, *L*. *kirschneri*, *L*. *borgpetersenii* and *L*. *mayottensis* with a specificity and reproducibility of 100%^12^. In other studies, HRM has been used to establish proof of principle assays for direct detection of *Leptospira* in human samples^14–16^. In a clinical context, qRT-PCR TaqMan and qRT-PCR-HRM has been used for diagnosis of leptospirosis using blood and/or urine samples^17–22^. However, none of these studies used paired human biological samples.

The aim of the present work was to implement a diagnostic assay for human leptospirosis capable of providing timely laboratory results on the same day the patient is seen at the emergency room of the hospital. To address this, we used a robust biobank of paired serum and urine samples collected from febrile patients at admission at the emergency room and evaluated the accuracy of qRT-PCR-HRM analysis as a clinical diagnostic tool for direct detection of *Leptospira* in the very early stages of human leptospirosis.

## Methods

### Ethical considerations

The present study followed international ethical guidelines and was evaluated and approved (Ref. HDES/CES/159/2009) by the Health Ethics Committee of the Hospital of Divino Espírito Santo of Ponta Delgada (HDES). The analysis of retrospective samples (serum and urine) from patients suspected of having leptospirosis was exempted from the need to obtain informed consent under the regulations of the Portuguese Data Protection Commission – law 12/2005 article 19, number 6 (https://www.cnpd.pt/bin/orientacoes/DEL227-2007-ESTUDOS-CLINICOS.pdf, accessed February 22, 2017).

### Study design

A total of 202 patients were investigated from January 2015 to June 2016 (Supplementary Table S1). Serum and urine were collected in sterile biological containers and processed for molecular detection/confirmation of *Leptospira* spp. We centrifuged all sera and urine samples at 2000 rpm for 10 minutes. Bacterial DNA was automatically extracted from 400 µl of independent samples of serum (S1 and S2) and urine (U1 and U2) from each patient using the BioRobot EZ1 Advanced System (Qiagen). A total of 808 samples were processed.

### Reference molecular test (conventional nested PCR)

Conventional nested PCR was considered the reference standard for *Leptospira* spp. DNA detection in the present study. After automatic bacterial DNA extraction, the *rrs* (16S rRNA) gene was amplified as previously described^23,24^ by conventional nested PCR in a Biometra^®^ T-Gradient thermal cycler. We used two primer sets: forward-A 5′-GGCGGCGCGTCTTAAACATG-3′ and reverse-B 5′-TTCCCCCCATTGAGCAAGATT-3′ for the first PCR; nested-A 5′-TGCAAGTCAAGCGGAGTAGC-3′ and nested-B 5′-TTCTTAACTGCTGCCTCCCG-3′ for the nested PCR. The first PCR reaction contained 5 μl of bacterial DNA, 10 μM primers A and B, 100 μM dNTPs (Promega), 25 nM MgCl_2_ (Qiagen), 1X Q-Solution (Qiagen), 1X buffer (Qiagen), 5 U of HotStart Taq (Qiagen) and RNase-free water to a final volume of 50 μl. The PCR programme started with an enzyme activation step at 95 °C for 15 minutes; proceeded with 30 cycles of 94 °C for 1 minute, 63 °C for 1 minute and 72 °C for 1 minute; and ended with a final extension step at 72 °C for 10 minutes. The nested PCR (2^nd^ round) used 5 μl of the first-round PCR product and 10 μM nested-A and nested-B primers. The first cycle consisted of denaturation at 95 °C for 15 minutes, followed by 30 cycles of denaturation at 94 °C for 1 minute, primer annealing at 63 °C for 1 minute, and extension at 72 °C for 1 minute, with an additional step at 72 °C for 10 minutes at the end, resulting in a 292 bp fragment. Amplified *Leptospira* DNA was visualized in an UV transilluminator instrument (BioRad) after agarose gel electrophoresis (3%). A patient was defined as having a laboratory-confirmed case of leptospirosis when *Leptospira* DNA was detected in at least one serum (S1 or S2) or urine (U1 or U2) sample.

### Real-Time PCR High Resolution Melting (qRT-PCR-HRM) analysis

Primer pairs for qRT-PCR-HRM analysis were chosen according to the results obtained by Naze *et al*.^12^. We used the following LFB1 F/R and G1/G2 primers to amplify the *lfb1* and *secY* genes, respectively: LFB1-F 5′-CATTCATGTTTCGAATCATTTCAAA-3′ and LFB1-R 5′-GGCCCAAGTTCCTTCTAAAAG-3′, and G1 5′-CTGAATCGCTGTATAAAAGT-3′ and G2 5′-GGAAAACAAATGGTCGGAAG-3′. The 15 μl reactions contained 7.5 μl of 2X Type-it HRM master mix (Qiagen), 0.7 μM final concentration of each primer (TibMolBiol), 3.75 μl of extracted bacterial DNA, and RNase-free water to a final volume of 15 μl. We performed the following amplification protocol in the 7500 Fast Real-Time PCR instrument (Applied Biosystems): denaturation at 95 °C for 5 minutes, followed by 45 cycles of 95 °C for 10 seconds, 55 °C for 30 seconds, and 72 °C for 10 seconds. These conditions were used for both primer sets. After PCR cycling, the samples were heated from 70 °C to 95 °C with continuous data acquisition.

We used six pathogenic *Leptospira* reference cultures provided by the Portuguese Reference Laboratory for Leptospirosis (at the Instituto de Higiene e Medicina Tropical, IHMT, of the Universidade Nova de Lisboa) as positive controls: 4 strains belonging to *L*. *interrogans* serogroup (sg) Icterohaemorrhagiae, *L*. *borgpetersenii* sg Ballum, *L*. *kirschneri* sg Cynopteri and *L*. *noguchii* sg Panama and 2 human Azorean isolates^4^ belonging to *L*. *interrogans* serovar (sv) Copenhageni of Icterohaemorrhagiae sg (human isolate 1) and *L*. *borgpetersenii* sv Arborea of Ballum sg (human isolate 6). Melting curve plots were generated and analysed using High Resolution Melt software v3.0.1 (Applied Biosystems) to determine average melting temperature (T_m_) for each *Leptospira* spp.

### qRT-PCR-HRM benchmarking confirmation by Sanger sequencing

To validate the qRT-PCR-HRM analysis, we selected 18 biological specimens (13 serum and 5 urine samples) from laboratory-confirmed leptospirosis patients, including the sample positive by nested PCR and negative by qRT-PCR-HRM analysis. As reference DNA sequences, we used two *Leptospira* spp. (*L*. *interrogans* sg Icterohaemorrhagiae and *L*. *borgpetersenii* sg Ballum) and two human isolates. Amplified DNA products of *Leptospira* obtained by nested PCR were purified using the QIAquick PCR Purification Kit (Qiagen) according to the manufacturer’s instructions. Sequencing was performed using the nested-A and nested-B primer pair with the BigDye Terminator v1.1 cycle sequencing kit (Applied Biosystems) under the following conditions: 2 μl of ready reaction mix, 4 μl of BigDye sequencing buffer, 3.2 pmol of each primer pair, 7 ng of DNA, and RNase-free water to a final reaction volume of 20 μl. The cycling programme included an initial denaturation step at 96 °C for 1 minute, followed by 25 cycles of 96 °C for 10 seconds, 50 °C for 5 seconds and 60 °C for 4 minutes, in a GeneAmp® PCR System 2700 (Applied Biosystems). The sequencing products were purified with a BigDye XTerminator® Purification Kit (Applied Biosystems) and separated by capillary electrophoresis in an automated sequencer (ABI 3130 Genetic Analyzer, Applied Biosystems) with a 36 cm capillary and POP-7™ polymer according to the manufacturer’s instructions. Data were analysed with Sequencing Analysis software v5.3.1 (Applied Biosystems). Sequences were aligned using Bioedit™ software v7.0.0.

### Microscopic agglutination test (MAT)

A total of 46 serum samples evaluated as positive by the molecular approach were aliquoted and stored at −20 °C for further detection of anti-*Leptospira* spp. antibodies by MAT. Additionally, 20 negative serum samples were selected as controls. MAT was performed at the Portuguese Reference Laboratory for Leptospirosis (IHMT, Universidade Nova de Lisboa) using a battery of 25 live pathogenic serovars (including 4 Azorean isolates) representative of 15 serogroups of pathogenic *Leptospira* and a saprophytic serovar of *L*. *biflexa* as an internal control. Samples were initially screened at a 1∶40 dilution, and reactive sera were further diluted in a 2-fold series to the endpoint, defined as the highest serum dilution that agglutinated at least 50% of leptospires. For the Azorean endemic region, samples were considered positive when titres were 1∶160 or greater, not conclusive when titres were below 1:160 (cut-off), and negative when no agglutination was observed.

### Statistical analysis

The nested PCR test was used as the reference molecular test to calculate the sensitivity, specificity, positive and negative predictive values (PPV and NPV), and overall accuracy [with the 95% confidence interval (CI)]. Calculations were performed using Vassar College’s VassarStats Website for Statistical Computation (http://www.vassarstats.net, last accessed May 16, 2018). To determine whether there was a significant difference between the diagnostic tests for *Leptospira* detection, data were analysed by McNemar’s test and by Fisher Exact test, *p* < 0.05 indicated statistical significance. The Standards for Reporting of Diagnostic Accuracy (STARD) statement was followed when reporting the results of the present study^25^.

### Data availability

All data generated or analysed during this study are included in this published article and the Supplementary Information files.

## Results

### Clinical presentation

The present work is a retrospective hospital-based study that includes samples from patients suspected of leptospirosis who presented mainly at the Emergency Department of the Hospital of Divino Espírito Santo of Ponta Delgada in São Miguel, Azores, a Portuguese island in which leptospirosis is endemic. A total of 202 patients were investigated from January 2015 to June 2016 (Supplementary Table S1). The mean patient age was 48.2 (±16.4) years. Higher rates of males (89.6%) than females (10.4%), farmers (20.3%) and unemployed persons (13.4%) were observed in the study population. Clinical diagnosis by the attending physician was based on signs and symptoms of leptospirosis, as previously described^23,26^. Briefly, physicians looked for epidemiological context, such as rural activities and direct contact with contaminated areas (rat urine), and clinical manifestations, including fever, myalgia, jaundice and coluria, before collecting biological samples (serum and urine) for molecular detection/confirmation of *Leptospira* spp. Analysis of admission records showed that 167 patients (82.7%) were seen at the emergency room and treated as outpatients, 18 patients (8.9%) were admitted to internal medicine and a7 patients (8.4%) were admitted to other departments.

### qRT-PCR-HRM assay

**Figure 1:**
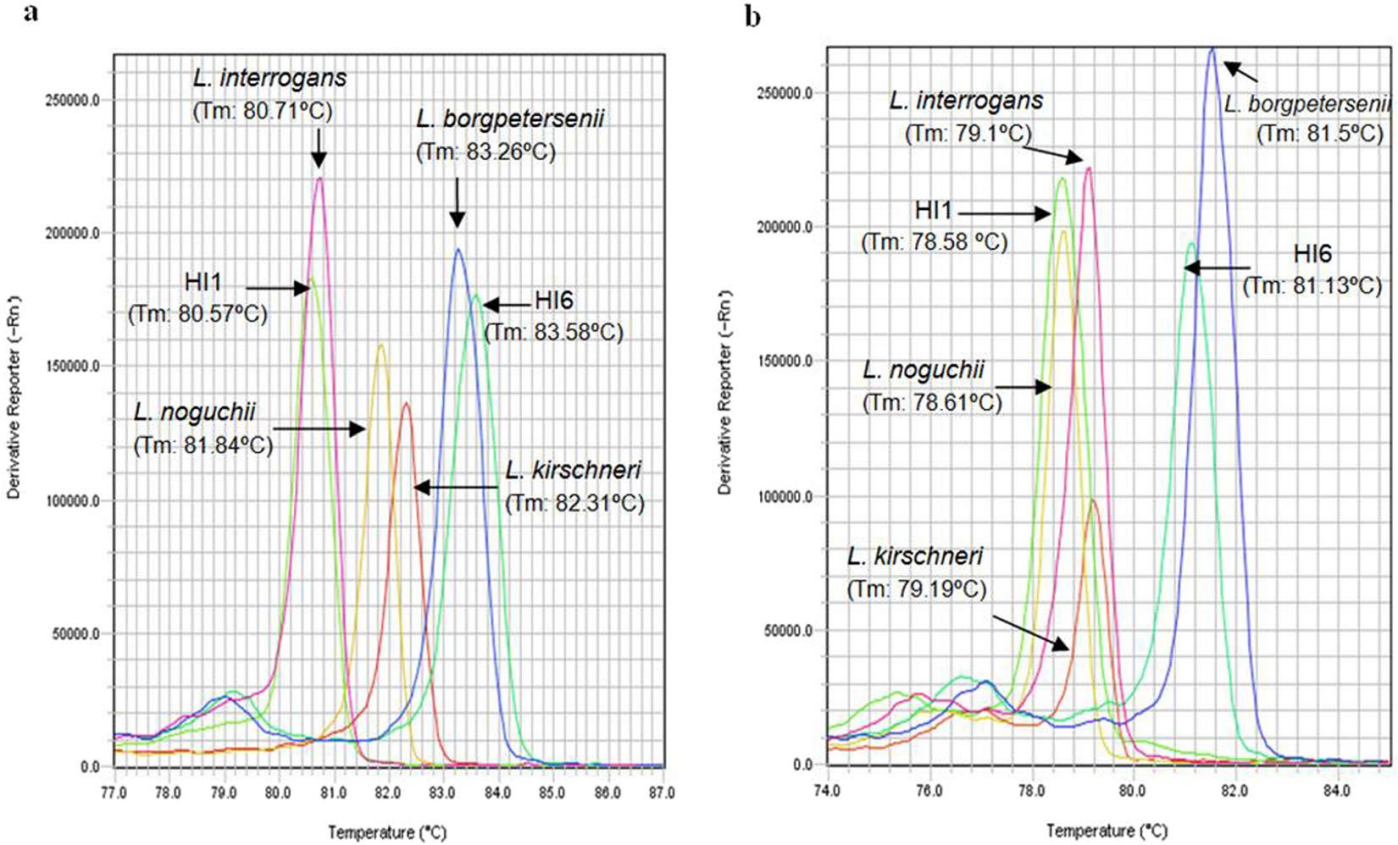
High resolution melting curve analysis profiles of cultured *Leptospira* spp., and *Leptospira* isolates from human leptospirosis patients. (**a**) HRM profiles using the LFB1 primer pair. (**b**) HRM profiles using the G1/G2 primer pair. Abbreviations: HI1, Human isolate 1; HI6, Human isolate 6.

The qRT-PCR-HRM assay was able to successfully distinguish 4 *Leptospira* spp. (*L*. *interrogans* sg Icterohaemorrhagiae, *L*. *borgpetersenii* sg Ballum, *L*. *kirschneri* sg Cynopteri and *L*. *noguchii* sg Panama) and the 2 human *Leptospira* isolates (HI1 and HI6). As shown in the derivative plot (Fig. 1), the LFB1 F/R and G1/G2 primer sets produced distinct melting curve profiles for reference *Leptospira* strains of *L*. *interrogans* and *L*. *borgpetersenii* spp. that matched those of the human *Leptospira* isolates (HI1 and HI6) of the same species. The T_m_ values obtained for LFB1 F/R were 80.71 °C (*L*. *interrogans)*, 81.84 °C (*L*. *noguchii*), 82.31 °C (*L*. *kirschneri*) and 83.26 °C (*L*. *borgpetersenii)*, and those for G1/G2 were 78.61 °C (*L*. *noguchii*), 79.10 °C (*L*. *interrogans)*, 79.19 °C (*L*. *kirschneri*) and 81.50 °C (*L*. *borgpetersenii)*. Moreover, these results were reproducible across 10 independent melt curve runs.

### qRT-PCR-HRM screening of samples from patients suspected of having leptospirosis

**Figure 2:**
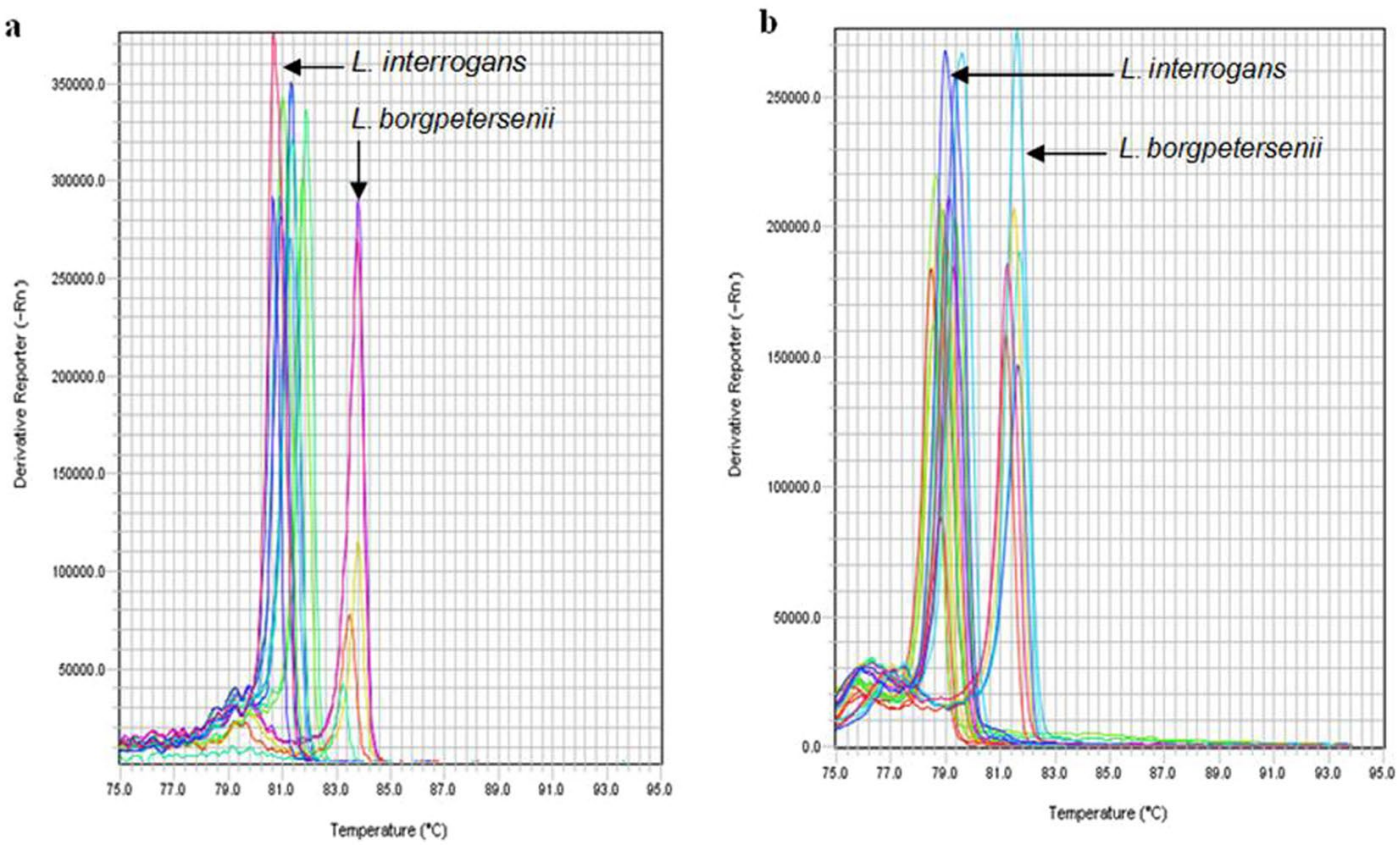
High resolution melting curve analysis profiles of human clinical samples (serum and urine) from patients with suspected leptospirosis. (**a**) HRM profiles using the LFB1 primer pair. (**b**) HRM profiles using the G1/G2 primer pair.

We screened 404 clinical specimens (paired serum and urine samples from 202 patients in duplicate, n = 808 samples) using qRT-PCR-HRM analysis. The average T_m_ with the LFB1 F/R primers was 80.94 °C in 28 (60.9%) and 83.84 °C in 16 (39.1%) patients (Supplementary Table S2). The average T_m_ with the G1/G2 primers was 79.36 °C in 28 (60.9%) and 81.82 °C in 18 (39.1%) patients. For both primer sets, we found one clinical sample to be positive by nested PCR and negative by qRT-PCR-HRM. The T_m_ obtained with *Leptospira* spp. and the melting curve profile results were consistent for the remaining patient samples (Fig. 2). We clustered the melting curves in two groups and identified the *Leptospira* spp. in the patient samples by comparing the T_m_ values to those of the six *Leptospira* positive controls.

### Kinetics of disease progression based on *Leptospira* detection

**Figure 3:**
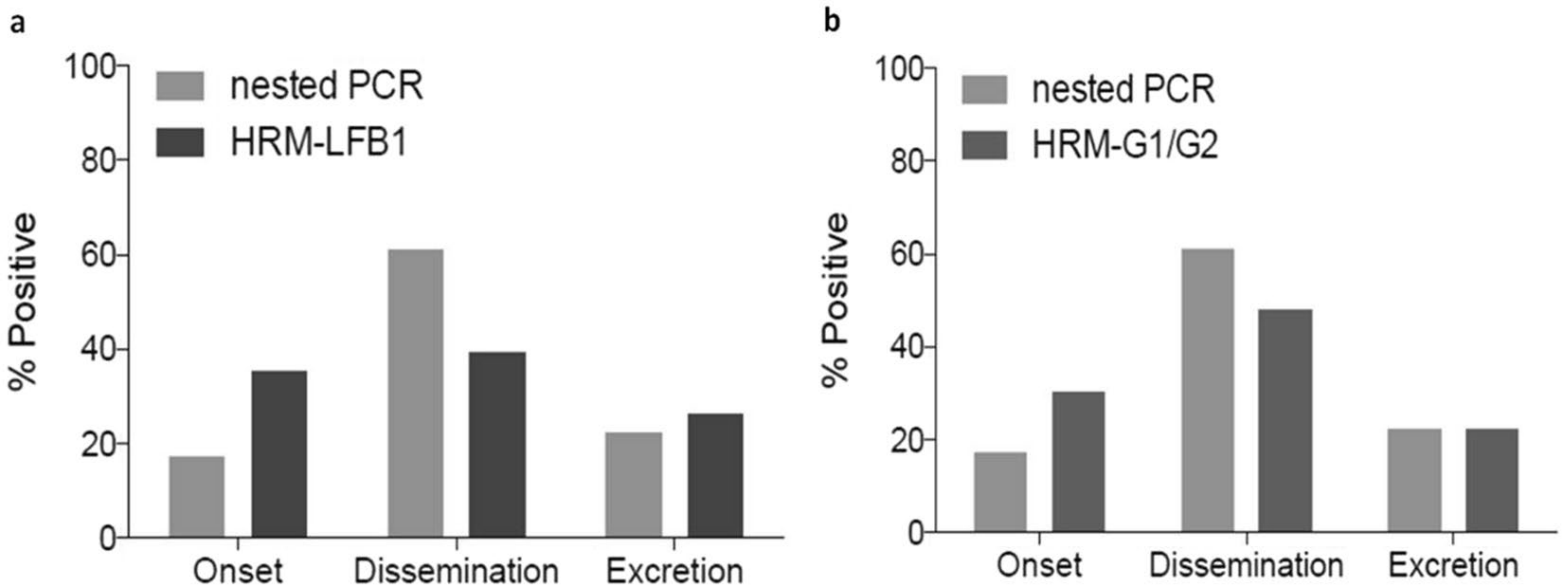
Sensitivity of molecular assays in function of the kinetics of disease progression. Comparative analysis of positive samples by nested PCR and qRT-PCR-HRM through the three early leptospirosis stages: onset (serum positive), dissemination and kidney colonization (serum and urine positive) and excretion (urine positive). (**a**) Comparison with primer pair LFB1. (**b**) Comparison with primer pair G1/G2. Statistics by Fisher Exact test, A *p* = 0.0033 and B *p* = 0.0763.

We evaluated paired serum and urine samples from 202 patients clinically suspected of having *Leptospira* spp. infection by nested PCR and qRT-PCR-HRM (Table 1). The nested PCR results were positive for 23.3% (47/202) patients and negative for 76.7% (155/202). Using qRT-PCR-HRM, the results were positive for 22.7% (46/202) patients and negative for 77.2% (156/202). The discrepant result between the two molecular assays was confirmed to be a false positive by sequencing (see below). qRT-PCR-HRM produced conclusive results in about half of the time (~2 hr) needed to generate nested PCR results (usually ~5 hr). Analysis of paired samples of the 46 laboratory-confirmed cases of leptospirosis suggested the following kinetics of disease progression (Table 2 and Fig. 3): samples which are positive only in serum (urine negative) represent patients undergoing early clinical onset of infection (incubation phase, Profile A), samples which are positive for serum and urine represent patients undergoing early dissemination and kidney colonization (early phase, Profile B) and samples which are only positive in urine (serum negative) represent patients that are excreting *Leptospira* (late phase, Profile C). A comparative analysis of these paired samples using both nested PCR and qRT-PCR-HRM-LFB1 primer pair showed that there is a redistribution of detection of positives between the onset and dissemination phases with nested PCR favouring dissemination (17% at onset and 61% at dissemination) and HRM gaining sensitivity at the onset of infection (35% at onset and 39% at dissemination). Differences are statistically significant by Fisher Exact test *p* = 0.0033. The same analysis comparing nested PCR and qRT-PCR-HRM-G1/G2 primer pair also showed a gain of sensitivity at the onset (17% to 30%, respectively), although differences in this case are not quite significant (Fisher Exact test *p* = 0.0763). The profile analysis also revealed that MAT highest percent positive was detected in the later excretion phase (serum negative and urine positive by molecular test) which is consistent with the kinetics of leptospirosis progression.

**Table 1:** Molecular characterization of 202 patients with suspected clinical leptospirosis.

Patients (N = 202)	Conventional nested PCR				qRT-PCR-HRM (primers)							
					LFB1				G1/G2			
	Positive		Negative		Positive		Negative		Positive		Negative	
	N	%	N	%	N	%	N	%	N	%	N	%
Total	47	23.3	155	76.7	46	22.7	156	77.3	46	22.7	156	77.3
**Duplicate samples (N = 808)**												
Serum												
S1	33	16.3	169	83.7	32	15.8	170	84.2	33	16.3	169	83.7
S2	34	16.8	168	83.2	26	12.9	176	87.1	30	14.9	172	85.1
Urine												
U1	35	17.3	167	82.7	27	13.4	175	86.6	30	14.9	172	85.1
U2	30	14.9	172	85.2	25	12.4	177	87.6	25	12.4	177	87.6

Duplicate serum and urine samples were investigated by conventional nested PCR and qRT-PCR-HRM methods.

**Table 2:** Molecular profiles of patients with laboratory-confirmed leptospirosis and the corresponding disease kinetics.

Kinetics of *Leptospira* infection			*Leptospira* detection methods							
			Conventional nested PCR (16S RNA)		qRT-PCR-HRM (primers)				MAT	
					LFB1		G1/G2			
Molecular profiles	Phases	Kinetics	N	%	N	%	N	%	N	%
Positive patients (after Sanger sequencing)			46	100	46	100	46	100	6	13.0
Profile A: Blood (serum+/urine−)	Incubation	Onset	8	17.4	16	34.8	14	30.4	0	0.0
Profile B: Blood and urine (serum+/urine+)	Early	Dissemination and kidney colonization	28	60.9	18	39.1	22	47.8	2	4.3
Profile C: Urine (serum-/urine+)	Late	Excretion	10	21.7	12	26.1	10	21.7	4	8.5

*P* > 0.05, conventional nested PCR compared with qRT-PCR-HRM; *P* < 0.0001, qRT-PCR-HRM compared with MAT.

### Sequencing analysis

**Figure 4:**
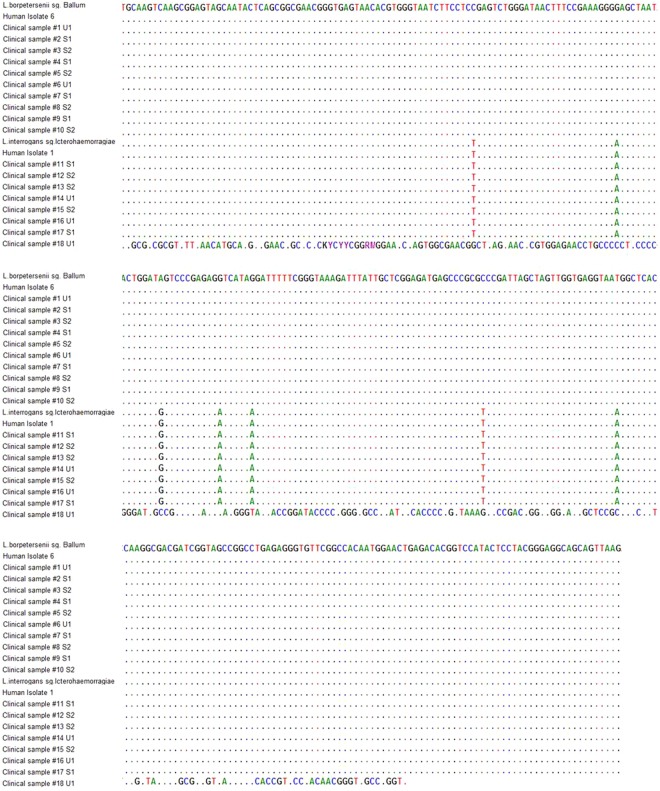
Confirmation of the qRT-PCR-HRM analysis by Sanger sequencing. Alignment of the consensus sequences of the clinical samples, *Leptospira* spp. and human isolates. Only the sequences showing differences from the first sequence are shown. Nucleotides identical to those in the first sequence are indicated by dots.

To benchmark *Leptospira* detection by qRT-PCR-HRM analysis, we performed Sanger sequencing (Fig. 4). The obtained bacterial DNA sequences confirmed the positive qRT-PCR-HRM results in 17 clinical samples. One sample (#18) was positive in the urine by nested PCR but negative by qRT-PCR-HRM. This sample was assessed twice by sequencing, qRT-PCR-HRM and nested PCR, and the sequence had a 97% match to *Collinsella aerofaciens*, which is found predominantly in the human gut. For the remaining samples (17/18), we observed a perfect match with the reference sequences regarding T_m_ values, melting curve profiles, and sequencing data.

### Microscopic agglutination test (MAT)

MAT results revealed that of the 46 nested PCR-positive patients, only 3 presented specific anti-*Leptospira* antibodies (6.5%), and 3 presented anti-*Leptospira* antibodies below the cut-off titre adopted by the Portuguese Reference Laboratory for Leptospirosis in the Azorean endemic region (Table 3).

**Table 3:** MAT results from the 46 patients with laboratory-confirmed leptospirosis.

Kinetics of *Leptospira* infection		Serovar	Species
Profiles	Conventional nested PCR (N = 46)	MAT	qRT-PCR-HRM
C	1	Positive (co-agglutination – highest titre 1:1280 against Arb A and Cyn)	*L*. *borgpetersenii*
	1	Positive (Arb A 1:160)	*L*. *borgpetersenii*
	1	Positive (co-agglutination – highest titre 1:320 against Arb A 1:320)	*L*. *interrogans*
	1	NC	*L*. *borgpetersenii*
	6	Negative	*L*. *interrogans*
B	1	NC	*L*. *interrogans*
	1	NC	*L*. *interrogans*
	16	Negative	*L*. *interrogans*
	10	Negative	*L*. *borgpetersenii*
A	3	Negative	*L*. *interrogans*
	5	Negative	*L*. *borgpetersenii*

Abbreviations: Arb A [serovar Arborea (Azorean isolate) serogroup Ballum]; Cyn [Cynopteri (reference serovar) serogroup Cynopteri]; NC, not conclusive (specific reactivity below the cut-off of 1:160 adopted in the Azorean endemic region).

### Analytical specificity and sensitivity of qRT-PCR-HRM

To validate the qRT-PCR-HRM analysis as a diagnostic method for *Leptospira* spp. detection, we assessed the accuracy parameters by comparing the results of nested PCR (reference molecular test) after sequencing with those obtained by qRT-PCR-HRM (Table 4). Of the 46 patients who were positive for leptospirosis by nested PCR, 46 had a positive qRT-PCR-HRM result, for a sensitivity of 1.00 (95% CI: 0.90–1.00). Moreover, of the 156 patients who were negative for leptospirosis by nested PCR, 156 had a negative qRT-PCR-HRM result, for a specificity of 1.00 (95% CI: 0.97–1.00). The PPV and NPV were 1.00 (95% CI: 0.90–1.00) and 1.00 (95% CI: 0.97–1.00), respectively. Overall, qRT-PCR-HRM accuracy was 100%. Together, these results confirm and validate the accuracy of qRT-PCR-HRM as a clinical diagnostic test for human leptospirosis.

**Table 4:** Diagnostic accuracy of qRT-PCR-HRM analysis compared with conventional nested PCR for detecting human leptospirosis infection.

Patients (N = 202)	qRT-PCR-HRM	
	Estimated value	95% CI
Sensitivity	1.00	0.90–1.00
Specificity	1.00	0.97–1.00
Positive predictive value (PPV)	1.00	0.90–1.00
Negative predictive value (NPV)	1.00	0.97–1.00
Accuracy (%)	100	—

Abbreviation: CI, confidence interval.

### Comparison between qRT-PCR-HRM and other qRT-PCR TaqMan chemistries

The current study is the first to present 100% accuracy values – specificity, sensitivity, positive predictive values (PPV) and negative predictive values (NPV) – for a molecular PCR method, validating the qRT-PCR-HRM as the best test to be implemented in a clinical setting (Table 4). Compared with qRT-PCR TaqMan chemistry, qRT-PCR-HRM has the highest diagnostic value (accuracy, affordability and time-to-result) making this test the best to inform treatment decisions for hospitalized patients and patients seen in emergency rooms or clinics (Table 5).

**Table 5:** Comparison between qRT-PCR-HRM (SYBR) and qRT-PCR TaqMan for diagnosis of leptospirosis in a clinical setting.

Patients (N) suspected of Leptospirosis	Clinical samples		*Leptospira*			Diagnostic accuracy				Country (geographic area)	Paper (year)
	Serum (N)	Urine (N)	Detection method	Molecular target	Nr. Positives (%)	Sensitivity	Specificity	Positive predictive value (PPV)	Negative predictive value (NPV)		
**202**	202*	202*	**qRT-PCR-HRM**	***lfb1*** **and** ***secy***	**46 (22.7)**	**100 (90–100)**	**100 (97–100)**	**100 (90–100)**	**100 (97–100)**	**Portugal** **(Azores)**	**Current study**
295	253 plasma	121	qRT-PCR-HRM MAT	*lipL32* *Ig*	15 (5.0)	60	100	100	100	Czech Republic	Cermakova Z. *et al*.^22^
235	235	0	qRT-PCR-HRM MAT	*lipL32* *Ig*	26 (11.0)	30	100	100	100	Uruguay	González S. *et al*.^19^
133	133	0	qRT-PCR-HRM	*secy*	26 (19.5)	100 (70–100)	100 (93–100)	NR	NR	Netherlands	Ahmed A. *et al*.^17^
266	133 blood	0	qRT-PCR TaqMan qRT-PCR TaqMan MAT Culture	*rrs PCR* *lipL32* *Ig* *Leptospira*	74 (55.6) 57 (42.8) 115 (86.4) 39 (29.3)	56 (47–64) 43 (34–52) 86 (79–92) 29 (22–38)	90 (83–94) 93 (88–97) 100 100	NR	NR	Thailand	Thaipadungpanit J. *et al*.^18^
787	785	644	qRT-PCR TaqMan qPCR MAT Culture	*rrs* *16S rRNA/LipL32* *Ig* *Leptospira*	76 (9.7) 20 (2.5) 30 (3.8) 4 (0.5)	50 (29.6–77.8) 53.9 (33.3–81.8) 15.8 (6.3–29.4) 25.0 (13.3–44.4)	99.2 (99–99.5) 99.6 (99.2–100) 96.5 (96.2–96.9) 100	57.1 (42.9–71.4) 75 (50–100) 10 (3.3–20) 100	99 (97.3–99.7) 99.1 (97.6–99.7) 98.3 (96.7–98.9) 98.5 (96.7–99.4)	Laos	Woods K. *et al*.^21^
150	150	0	qRT-PCR TaqMan	*lipL32*	127 (84.6)	29.1 (21.6–38.0)	99	NR	NR	Brazil (Salvador and Curitiba)	Riediger I. N. *et al*.^33^

*Analysed in duplicate; NR, not reported.

## Discussion

In this study, real-time PCR-HRM assay was validated for the accurate detection of *Leptospira* DNA using 202 paired biological samples from patients presenting in the emergency room of a hospital in the Azorean island of São Miguel, a Portuguese region endemic for leptospirosis. Among 202 human patients suspected of having leptospirosis, 46 tested positive (22.7%) by both nested PCR and qRT-PCR-HRM; among the suspected patients, only 3 tested positive (6.5%) by MAT. Melting curve profiles with the LFB1 F/R primer set distinguished the 4 *Leptospira* spp., *L*. *interrogans*, *L*. *borgpetersenii, L*. *kirschneri* and *L*. *noguchii*, in cultured bacteria and human isolates (Fig. 1). These results are in accordance with those obtained by Naze and collegues^12^. In addition, template-independent amplifications targeting the two relevant genes (*lfb1* and *secY*) of pathogenic *Leptospira* spp. also provided a thorough validation of the present qRT-PCR-HRM assay. The 404 human samples used – paired serum and urine from 202 patients, analysed in duplicated (total of 808 specimens) – validate the application of qRT-PCR-HRM as a clinical diagnostic test for human leptospirosis in a clinical setting.

The melting curve analysis of *Leptospira* species in patient samples (serum and urine) accurately discriminated species when positive controls were included in each run (Fig. 2). According to the T_m_, the qRT-PCR-HRM assay revealed that 60.9% (28/46) of patients were infected with leptospires belonging to *L*. *interrogans*, and 39.1% (18/46) were infected with leptospires belonging to *L*. *borgpetersenii* species (Supplementary Table S2). The most likely explanation for these results is that *L*. *interrogans* survives longer when exposed to the environment, which is why it is more prone than *L*. *borgpetersenii* to infect humans. The latter does not survive in the environment and is transmitted by direct contact with the host^27^. MAT is the hundred-year old gold standard method for the serodiagnosis of leptospirosis and allows for the determination of the presumptive serogroup or serovar of the infecting strain in routine diagnostics and/or epidemiological studies^28^. In the present study, MAT results substantiated the qRT-PCR-HRM findings, as these patients presented anti-*Leptospira* antibodies belonging to one of these serogroups. In addition, MAT results were positive in only 3/46 (6.5%) of the HRM-positive samples which is expected in recently infected febrile patients and explained by the typical delay period between time of infection and presence of measurable levels of antibodies in blood. Low MAT sensitivity in an early stage of disease infection was discussed previously^29^. In a clinical diagnostic context, this observation alone qualifies qRT-PCR-HRM as a valuable alternative to MAT by providing early unambiguous diagnosis of the disease, which can better inform treatment decisions by the physician as recommended by WHO^30^. The qRT-PCR-HRM method validated in the present study not only detects *Leptospira* in human biological samples with 100% accuracy, but also informs epidemiology of the disease by identifying the infecting species.

By conducting DNA sequencing as part of the assay validation, we determined that the leptospires infecting these patients belonged to the serogroups Icterohaemorrhagiae and Ballum (Fig. 4). These results agree with prior studies performed in the Azores Islands (São Miguel and Terceira), where the serogroups Icterohaemorrhagiae (*L*. *interrogans*) and Ballum (*L*. *borgpetersenii*) were the most frequent human^23,26,31^ and rodent *Leptospira* isolates^2,32^.

The profiles based on the 46 confirmed positive patients (by nested PCR and qRT-PCR-HRM) described in Table 2 are consistent with the kinetics of *Leptospira* infection and disease progression in humans^3,27^. The analysis allows us to identify the illness point at which patients presented at the hospital. Infection produces leptospiraemia within the first days after exposure, which is followed by the appearance of leptospires in multiple organs by the 3^rd^ day of infection (incubation period and dissemination). Illness develops with the appearance of agglutinating antibodies 5–14 days after exposure (early phase). Leptospires are cleared from the bloodstream and organs in the late phase, as serum agglutinating antibody titres increase^27^. A higher percentage of patients in this study were seen in the early phase of the disease (Profile B, Table 2), when the immune system starts to produce antibodies and clearing *Leptospira* from the blood, which is why the bacteria is detected in serum and urine. Another important finding is that qRT-PCR-HRM is more sensitive than nested PCR at detecting *Leptospira* during the incubation period (first seven days, Profile A, Fig. 3). This finding is of clinical relevance because it allows for the immediate initiation of antibiotic therapy at the earliest onset. Regarding Profile C, 23.4% of patients presented at the hospital when *Leptospira* DNA is detected in the urine. This delay in coming to the hospital probably occurs because the symptoms are similar to those of flu, and patients stay at home and take conventional over-the-counter medicine. For patients with Profile C, qRT-PCR-HRM was more specific than nested PCR; one patient was positive by nested PCR and negative by HRM. Bacterial DNA sequencing of this patient’s urine sample (#18) showed a 97% match to *Collinsella aerofaciens*, a type of bacteria found in the human gut, proving that the nested PCR result was a false positive. This finding highlights the caution necessary when interpreting the results of assays such as nested PCR that target the *rrs* gene (encoding 16S rRNA), which is conserved among many bacterial species, and are thus prone to cross-reactivity^19^. The performance of the qRT-PCR-HRM assay was evaluated and compared with that of the reference molecular test, nested PCR (Table 4); qRT-PCR-HRM was 100% accurate. The high specificity (100%) and sensitivity (100%) of qRT-PCR-HRM in endemic regions, such as the Azores, is highly relevant. Notably, since the nested PCR technique was implemented at HDES in 2005, no patient on São Miguel Island has died of leptospirosis. According to official data in the Azores (the islands of São Miguel and Terceira) for the period between 1986 and 2002, fewer than 19 deaths due to leptospirosis were reported each year^31^.

In clinical diagnostic laboratories, real-time PCR methods (SYBR green and TaqMan chemistries) are increasingly being used instead of conventional PCR methods, providing the opportunity to rapidly confirm leptospirosis infection in the first days of infection. The qRT-PCR-HRM allows for accurate clinical diagnosis of leptospirosis in just 2 hours, rather than the 5 hours needed for nested PCR, the results are unambiguous and easy to interpret and due to the use of SYBR green the costs are far below those needed for TaqMan chemistry. The HRM assay is a robust molecular PCR method for the diagnosis of human leptospirosis infection in endemic regions and it can be fully implemented in routine clinical laboratories with real-time PCR equipment. Furthermore, qRT-PCR-HRM has the advantage of allowing for the distinction of *Leptospira* species which informs leptospirosis epidemiology in the geographic region without requiring the maintenance of large strain collections and labourious cultures. Two studies need to be highlighted as they tested several biological samples from more than 100 patients. In Cermakova *et al*.^22^ of the 295 suspected patients tested, 5% were positive by real-time PCR. In that study positive samples were assigned to urine, plasma, and CSF. Although qRT-PCR-HRM was described, it is not clear if it was used to classify positive and negative samples. Woods *et al*.^21^ did the most comprehensive study to date on diagnosis of leptospirosis using real-time PCR in blood and urine samples from 778 patients. In that study they found that ~10% of patients were positive by *rss* qPCR and 2.5% were positive by the more specific 16 s rRNA/LipL32 qPCR, in comparison with 3.8% patients detected by MAT and 0.4% by culture. Furthermore, they found that serum and urine are the best samples for qPCR and that quantitative PCR on hospital admission is a rapid and reliable diagnostic tool that performs better than MAT or culture. Our study in the Azores substantiates Woods’ study in Laos, and adds the following novelty to the field: (1) only in our study a paired analysis of serum and urine from the same patient was done; (2) because prevalence of leptospirosis in the Azores, Portugal was 2x to 10x higher (~23%) than Woods reports for Laos (~2.5 to ~10%) analysis of our paired samples allowed us to identify the illness point at which patients presented at the hospital. Another difference is that we did melting curve analysis which allowed us to determine that the assay is more sensitive for diagnosis of leptospirosis at the very early incubation phase (serum positive, urine negative) and we were able to identify the infecting species; all of this was done under 2 hours.

In conclusion, we did a unique comparative analysis using a robust biobank of paired samples of serum and urine from the same patient to validate the qRT-PCR-HRM assay for molecular diagnosis of human leptospirosis in a clinical setting. Furthermore, rapidly distinguishing *Leptospira* species while performing a rapid diagnostic test adds an epidemiological advantage to the assay over current clinical molecular diagnostic techniques.

## Electronic supplementary material

Supplementary information
