# Supplementary material for: Diagnosis of Human Leptospirosis in a Clinical Setting: Real-Time PCR High Resolution Melting Analysis for Detection of Leptospira at the Onset of Disease
Source: Sci Rep. 2018 Jun 15;8:9213. doi: 10.1038/s41598-018-27555-2 (PMC6003994; doi:10.1038/s41598-018-27555-2)
Supplement: Supplementary file 1 — Supplementary information [file 41598_2018_27555_MOESM1_ESM.pdf]

SUPPLEMENTARY INFORMATION

Diagnosis of Human Leptospirosis in a Clinical Setting: Real-Time PCR High Resolution Melting Analysis for Detection of *Leptospira* at the Onset of Disease

Lisa M. Esteves[1], Sara M. Bulhões[1], Claudia C. Branco[1,2,3], Teresa Carreira[4], Maria L. Vieira[4], Maria Gomes-Solecki[5] & Luisa MotaVieira[1,2,3*]

[1]Molecular Genetics and Pathology Unit, Hospital of Divino Espírito Santo of Ponta Delgada, EPER, São Miguel Island, Azores, Portugal. [2]Azores Genetics Research Group, Instituto Gulbenkian de Ciência, Oeiras, Portugal. [3]Centre for Biodiversity, Functional and Integrative Genomics, Faculty of Sciences, University of Lisboa, Portugal. [4]Global Health and Tropical Medicine (GHTM), Instituto de Higiene e Medicina Tropical (IHMT), Universidade Nova de Lisboa (UNL), Portugal. [5]Department of Microbiology, Immunology and Biochemistry, University of Tennessee Health Science Center, Memphis, Tennessee, USA.

[*] Correspondence to Luisa Mota-Vieira (Luisa.MQ.Vieira@azores.gov.pt)

Demographic characterization	Patients	
	N = 202	%
**Age at infection (yr)**		
Mean	48.2±16.4	–
Range	17-86	–
**Gender (n, %)**		
Male	181	89.6
Female	21	10.4
**Profession (n, %)**		
Farmer	41	20.3
Unemployed	27	13.4
Bricklayer	26	12.9
Retired	24	11.9
Housewife	12	5.9
Students	11	5.5
Federal employee	7	3.5
Businessman	6	3.0
Factory worker	4	2.0
Others	44	21.8
**Municipalities of São Miguel Island (n, %)**		
Ponta Delgada	110	54.5
Ribeira Grande	43	21.3
Lagoa	15	7.4
Vila Franca do Campo	15	7.4
Povoação	11	5.5
Nordeste	6	3.0
Santa Maria Island (n, %)	2	1.0
**Hospital department (n, %)**		
Emergency	167	82.7
Internal medicine inpatients	18	8.9
Others	17	8.4

Table S1. Demographic characterization of the 202 patients suspected of leptospirosis.

Patients		qRT-PCR-HRM primers (average T_m ± SD, ºC)		
**N = 46**	**%**	**LFB1 F/R**	**G1/G2**	***Leptospira* spp**
28	60.9	80.94 ± 0.27	79.36 ± 0.26	*L. interrogans*
18	39.1	83.84 ± 0.27	81.82 ± 0.30	*L. borgpetersenii*

Table S2. Determination of the melting temperatures (Tm), by qRT-PCR-HRM with primer sets LFB1 F/R and G1/G2. The assay was performed in the 46 patients with laboratory-confirmed leptospirosis (corresponding to 184 duplicate samples of serum and urine). Abbreviation: SD, standard deviation.
